# Influence of parenting style on the self-esteem of adolescents and the factors associated with low self-esteem: An institutional based cross-sectional study in Tokha municipality, Nepal

**DOI:** 10.1371/journal.pone.0347664

**Published:** 2026-05-13

**Authors:** Pratibha Bhandari, Prakash Adhikari, Rejina Baruwal, Kumar Bolakhe, Shanti Prasad Khanal, Niraj Bhattarai, Samip Khatri, Dinesh Neupane

**Affiliations:** 1 Central Department of Public Health, Institute of Medicine, Tribhuvan University, Kathmandu, Nepal; 2 Nepal Development Society, Kathmandu, Nepal; 3 University of Illinois Urbana-Champaign, Champaign, United States of America; 4 Faculty of Education, Tribhuvan University, Kathmandu, Nepal; 5 Department of International Health, Johns Hopkins Bloomberg School of Public Health, Baltimore, Maryland, United States of America; Fallujah University, IRAQ

## Abstract

Adolescence is a crucial developmental phase where self-esteem significantly impacts mental health and life outcomes. Low self-esteem is associated with depression, academic struggles, and risky behaviors. Parenting style plays a key role in shaping self-esteem, though its effects vary across cultural contexts. Despite robust global evidence, empirical research on parenting style and adolescent self-esteem in Nepal remains limited. This study addresses this gap by examining the association between perceived parenting styles, socio-demographic factors, and self-esteem among adolescents in urban Nepal. This cross-sectional study was conducted among 343 adolescent students in grades 9 and 10 in the schools of Tokha municipality using probability sampling. Self-esteem and perceived parenting styles were assessed by using the Rosenberg Self-Esteem Scale and the Perceived Parenting Style Scale. Data was analyzed using descriptive statistics, univariable and multivariable logistic regression, and spearman correlation at a significance level of 0.05 to identify factors associated with low self-esteem. The mean age (±S.D.) of the respondents was 15.3 (±1.0) years. One in six adolescents (16.3%) had low self-esteem. Authoritative parenting was positively correlated with self-esteem (r = 0.33, p < 0.01), whereas authoritarian (r = −0.32, p < 0.01) and permissive (r = −0.25, p < 0.01) parenting showed negative correlations. Older students were less likely to have low self-esteem (aOR: 0.7; 95% CI: 0.5–0.9). Conversely, students from private schools (aOR: 2.1; 95% CI: 1.1–4.2) and those whose fathers had less than a secondary education (aOR: 3.8; 95% CI: 1.9–7.4) were more likely to have low self-esteem. The major limitations of this study are the lack of representativeness in rural context, inability to address potential confounders and social desirability bias. Our findings suggest that authoritative parenting fosters higher self-esteem in adolescents, while authoritarian and permissive styles have negative effects. Future interventions should target younger adolescents, students from private schools, and those with fathers with lower educational attainment to promote healthy self-esteem development. Similarly, programs targeting parents to improve their knowledge, attitude and skills to practice authoritative way of parenting should be implemented.

## Introduction

Adolescence is a critical developmental phase of life characterized by significant physical, emotional, and social changes. These changes often mark unique pressures and stressors that can profoundly impact psychological well-being among the adolescents [1]. According to the World Health Organization (WHO), investing in adolescent health yields a “triple dividend” of benefits by benefiting adolescents today, shaping their future adult lives, and influencing the next generation [2]. Consequently, healthy and resilient adolescents are essential for creating sustainable and prosperous societies [2].

Self-esteem, defined as an individual’s subjective opinion of their own value, plays a pivotal role in adolescent mental health. Higher self-esteem is associated with greater psychological well-being, while low self-esteem has been linked not only to poor physical health but also to adverse outcomes such as depression [3,4], substance abuse [5], criminal behaviors, poor health outcomes [6], risky sexual behaviors [7], and even suicidal behavior [8,9]. Alarmingly, WHO reported that Nepalese adolescents experience one of the highest rates of suicidal ideation (14%) and suicidal attempts (10%) in South-East Asian region [10]. Moreover, low self-esteem of Nepalese adolescents has been reported around a range of 21% to 29% according to previous studies [8,10–13], which highlights an urgent need for targeted interventions in this area. Furthermore, there might be various factors associated with the low level of self-esteem among Nepalese adolescents which need to be explored as the extant literature on this area is mainly descriptive rather than analytical.

Parenting style, the emotional and disciplinary climate parents create for their children, is a critical factor shaping adolescents’ psychological well-being [14]. Baumrind introduced three primary types of parenting styles—authoritative, authoritarian, and permissive [15]. According to it, authoritative parenting emphasizes warmth, clear expectations, and mutual respect, fostering positive outcomes for children. In contrast, authoritarian parenting relies on strict rules and punitive measures, often with limited communication. Permissive parenting, characterized by leniency, minimal demands and low level of supervision, provides children with freedom but may lack the structure necessary for healthy development [16]. In Nepal, all three parenting styles are in practice to certain extents, albeit authoritative parenting is reported as being the most prevalent in the urban area of Nepal [13,17].

Bronfenbrenner’s Ecological Systems Theory highlights the role of multiple environmental contexts in shaping adolescent development, with particular emphasis on parents and family within the bioecological model of human development [18]. Based on this theoretical perspective, it is reasonable that parents’ understanding of adolescent self-esteem development, their daily parenting practices, and their overall parenting style are closely associated with adolescents’ self-esteem development, albeit the strength and direction of this association may vary across cultural and socioeconomic contexts.

Internationally, various studies in the United States [19], parts of Europe such as Hungary [20] and Spain [21] have highlighted the benefits of authoritative parenting, whereas permissive parenting was found to be optimal for child-rearing in other parts of the world like Brazil and India [22,23], indicating that the association between parenting style and self-esteem association is not uniform across all parts of the world, consequently highlighting the necessity of examining their association within specific cultural and contextual frameworks for better planning and implementation of need-specific interventions and programs for adolescents.

However, despite growing international evidence, research examining the link between parenting styles and adolescent self-esteem in Nepal remains scarce. There are only a few studies in Nepal that assess the types of parenting styles and even fewer that have examined the association between parenting style and self-esteem of adolescents. Similarly, urban Nepal is undergoing rapid social, cultural, and economic changes that are reshaping family dynamics and parenting practices. Increased exposure to globalization, academic competition, digital media, shifting gender norms, and evolving household structures may influence how parents interact with adolescents and how adolescents develop self-esteem. Understanding this association in the Nepali urban context is essential for identifying psychosocial risk and protective factors, informing mental health and school-based interventions, and guiding parents and policymakers in supporting adolescent emotional well-being. This study aims to examine the relationship between perceived parenting styles and self-esteem among adolescents and the factors associated with the low self-esteem in urban region of Nepal.

## Materials and methods

### Study design

An institutional based cross-sectional study design was employed to assess the association of self-esteem of adolescents with perceived parenting styles, and identify the factors associated with low self-esteem.

### Setting of the study

The study was conducted in one of the major municipalities of Kathmandu district, namely Tokha, which has one of the highest numbers of adolescent school students among all municipalities in Nepal [24]. This site was selected based on the number of adolescents, number of schools and population density, as people from different places of Nepal migrate here to provide better educational opportunities for their children, consequently making the sample population more inclusive and representative. The recruitment and data collection were done from 26^th^ January to 10^th^ February 2024.

### Study participants

The study population consisted of all the students studying in grades 9 and 10 among the randomly selected two private and two public schools of Tokha municipality. The inclusion criteria included all the students aged 10–19 currently studying in grade 9 or 10 of the selected schools, who lived with one or both of their parents. Students who didn’t have at least one parent, lived in a hostel and/or were absent during the days of data collection were excluded.

### Sampling technique and sample size

Simple random probability sampling was used for this study. A total of 81 schools were listed in Tokha Municipality based on records obtained from the Education Section of Tokha Municipality. Initially, four wards were selected randomly from the eleven wards of the municipality. From these selected wards, all private schools were categorized as Category A and all public schools as Category B. Using the lists provided by the Education Section, two private and two public schools were randomly selected from the respective categories through a lottery method. Subsequently, all sections of classes 9 and 10 from each selected school were included in the study, and all students from these sections were taken as study participants.

The expected proportion in population was taken as 29.8% from a similar study conducted in Nepal [10] and the sample size was calculated using the formula n = z^2^pq/e^2^, where *p* = 0.298, q = 0.702, z = 1.96 at 95% confidence interval, and e = 0.05 [25]. The resultant sample size was 322 from the above formula. Assuming 10% as the non-response rate, the sample size of 358 was estimated. We adopted standardized scales to collect the data.

### Data collection tools and technique

Data was collected using a paper-based, self-administered, close-ended questionnaire. Each student provided a single response under direct observation of the research assistants. Self-esteem was measured using the Rosenberg Self-Esteem Scale (RSES), a 10-item, 4-point Likert scale (0–3). Items 2, 5, 6, 8, and 9 were reverse scored, with a total score of 30 [26]. The English version of RSES questionnaire demonstrated excellent reliability (Guttman coefficient = 0.92, test-retest r = 0.85–0.88) and strong validity [26]. Similarly, perceived parenting style was assessed using the Perceived Parenting Style Scale by Divya and Manikandan [27]. This validated tool consists of a 5-point Likert scale (1–5) with 10 items for each of the three parenting styles. Scores were summed for each style. The English version of this scale has high internal consistency (Cronbach’s alpha = 0.79–0.86) and face validity [27].

The questionnaire was first drafted in English language and was later translated into Nepali language. A rigorous translation process was conducted to ensure the scale’s appropriateness. Translation in Nepali language was undertaken by the principal investigator who was proficient in both source and target languages. Also, an independent translator who was unfamiliar with the original scale, conducted a back translation of the entire questionnaire, including socio-demographic section, RSES scale and PPSS scale.

### Measurements

#### Dependent variable.

Self-esteem was assessed using the RSES questionnaire, which yields scores ranging from 0 to 30. Based on previous studies conducted among students, the scores were dichotomized into low self-esteem (<15) and medium-to-high self-esteem (15–30) [10].

#### Independent variables.

Perceived parenting style was the main independent variable measured using Perceived Parenting Style Scale. Socio-demographic variables included age (in completed years), sex (male, female), school type (private, public), family type (nuclear, joint and extended), religion (Hindu, Buddhist, Christian, Muslim, Others), parents’ marital status (married and living together, married and not living together, widowed, divorced and separated), highest educational level of parents (illiterate, literate (no formal schooling), primary level, secondary level, higher secondary level and university level), ethnicity (Brahmin/Chhetri, Janajati, Madhesi, Muslim, Dalit, and others, based on the Health Management Information System (HMIS) classification of the Government of Nepal [28]. The academic year of the students was categorized into grade 9 and grade 10. GPA was measured as GPA score in the most recent final exams taken by the students and categorized as (Below good if GPA < 2.40, Good and above if GPA ≥ 2.40)

### Data management and statistical analysis

Data entry was done in Microsoft Excel and data cleaning, and analysis was done using Stata statistical software (version 18). Of the 355 responses collected, 12 were excluded due to incomplete data, leaving 343 for analysis.

Normality of the dependent variable was assessed using the Kolmogorov–Smirnov test and histogram. As the data were not normally distributed, non-parametric tests were applied. Descriptive statistics were used to summarize socio-demographic characteristics (mean and standard deviation for continuous variables; frequency and percentage for categorical variables). Associations between categorical variables and self-esteem levels were examined using the Chi-square test and Fisher’s exact test. Correlations between continuous variables (parenting styles) and self-esteem were assessed using Spearman’s rank correlation. Variables with p < 0.1 in univariate logistic regression were included in the multivariable logistic regression model. Statistical significance was set at p ≤ 0.05.

### Ethical considerations

Ethical approval for the research was obtained from the Institutional Review Committee of the Institute of Medicine (IOM), Tribhuvan University, Nepal (Reference no: 308(6–11) E2 080/081). Then, the permission for data collection was obtained from the Tokha municipality education section (Reference no: 080-081-1338). Following that, the permission for data collection was obtained from the respective schools. Respondents were informed about the study’s aims. Written consent was obtained from all participants. Similarly, parental consent followed by their children’s written assent was taken for those under 18. To ensure privacy and confidentiality, no identifiers were included in the questionnaire.

## Results

### Socio-demographic characteristics

A total of 343 participants were included in the final analysis. Males (50.7%) were almost equal in proportion to females (49.3%). The mean age (± S.D.) of the respondents was 15.3 (±1.0) years. Brahmin/ Chhetri (49.3%) was the major ethnic group followed by Janajati (40.2%), Dalit (5.0%) and others (5.5%).Most of the respondents followed Hinduism as their religion (83.1%). Majority of the respondents belonged from nuclear family (54.5%). In terms of parental education, fathers were found to have higher educational status than mothers of the participants. 17.9% of participants’ mothers were illiterate, which was almost three times higher than the prevalence of illiteracy among fathers (6.6%). On the other end, a somewhat similar percentage of mothers and fathers had completed secondary and above level of education, at 54.5% for mothers and 66% for fathers of respondents. Regarding academic performance, about one in eight students had a GPA below good level. Likewise, around one in six (16.3%) respondents had low self-esteem. The respondent’s age, type of school and father’s education level were significantly associated with the level of self-esteem. (p ≤ 0.05). Adolescents belonging to younger age groups (13–15) were found to have significantly lower self-esteem (20.1%) than those belonging to older age groups (16–18). Those adolescents whose fathers had no formal schooling reported having significantly lower self-esteem (33.3%) compared to those whose fathers had secondary and higher level of educational background (Table 1).

**Table 1:** Characteristics of the participants (n = 343).

Variables	Categories	N (%)	Self-esteem Level		p-value
			Low	Normal-high	
Total Participants		343 (100.0)	56 (16.3)	287 (83.7)	
Age	13-15	199 (58.0)	40 (20.1)	159 (79.9)	0.03
	16-18	144 (42.0)	16 (11.1)	128 (88.9)	
Sex	Male	174 (50.7)	24 (13.8)	150 (86.2)	0.19
	Female	169 (49.3)	32 (18.9)	137 (81.1)	
School Type	Private	126 (36.7)	27 (21.4)	99 (78.6)	0.05
	Public	217 (63.3)	29 (13.4)	188 (86.6)	
Ethnicity	Brahmin/Chhetri	169 (49.3)	23 (13.6)	146 (86.4)	0.37
	Janajati	138 (40.2)	27 (19.6)	111 (80.4)	
	Others	36 (10.5)	6 (16.7)	30 (83.3)	
Religion	Hindu	285 (83.1)	47 (16.5)	238 (83.5)	0.86
	Others	58 (17.9)	9 (15.5)	49 (84.5)	
Father’s Education (n = 335)	Illiterate	22 (6.6)	2 (9.1)	20 (90.9)	<0.01
	Literate (No formal schooling)	48 (14.3)	16 (33.3)	32 (66.7)	
	Primary level	44 (13.1)	10 (22.7)	34 (77.3)	
	Secondary level	88 (26.3)	8 (9.1)	80 (90.9)	
	Higher secondary level	76 (22.7)	8 (10.5)	68 (89.5)	
	University level	57 (17.0)	11 (19.3)	46 (80.7)	
Mother’s Education (n = 341)	Illiterate	61 (17.9)	9 (14.8)	52 (85.3)	0.94
	Literate (No formal schooling)	62 (18.2)	10 (16.1)	52 (83.9)	
	Primary level	32 (9.4)	6 (18.8)	26 (81.3)	
	Secondary level	85 (24.9)	13 (15.3)	72 (84.7)	
	Higher secondary level	65 (19.0)	10 (15.4)	55 (84.6)	
	University level	36 (10.6)	8 (22.2)	28 (77.8)	
GPA (n = 335)	Below Good	40 (11.9)	8 (20)	32 (80)	0.48
	Good and above	295 (88.1)	46 (15.6)	249 (84.4)	
Family Type	Nuclear	187 (54.5)	37 (19.8)	150 (80.2)	0.06
	Joint/ Extended	156 (45.5)	19 (12.2)	137 (87.8)	
Parent’s Marital Status*	Married	326 (95.0)	53 (16.3)	273 (83.7)	0.88
	Separated/Widow/Widower	17 (5.0)	3 (17.7)	14 (82.4)	

Data analyzed using χ² test, *Fischer Exact Test.

### Perceived parenting styles of the participants

Table 2 displays the distribution of perceived parenting styles of the participants. The median score for authoritative parenting was 43 (IQR: 37–46) indicating that most parents exhibited authoritative parenting behaviors with their children. Similarly, authoritarian parenting type’s median score was 21 (IQR: 17–26) which suggests the relatively lower prevalence of this practice. Contrarily, the median of permissive parenting was quite low, at 14 (IQR: 11–18) implying that this parenting practice was unpopular among the participants’ parents (Table 2).

**Table 2:** Perceived Parenting Styles (n = 343).

Variables	Median (Q1–Q3)	Min–Max
Authoritative Parenting	43 (37-46)	21-50
Authoritarian Parenting	21 (17-26)	11-41
Permissive Parenting	14 (11-18)	10-47

### Self-esteem level distribution among the participants

**Fig 1:**
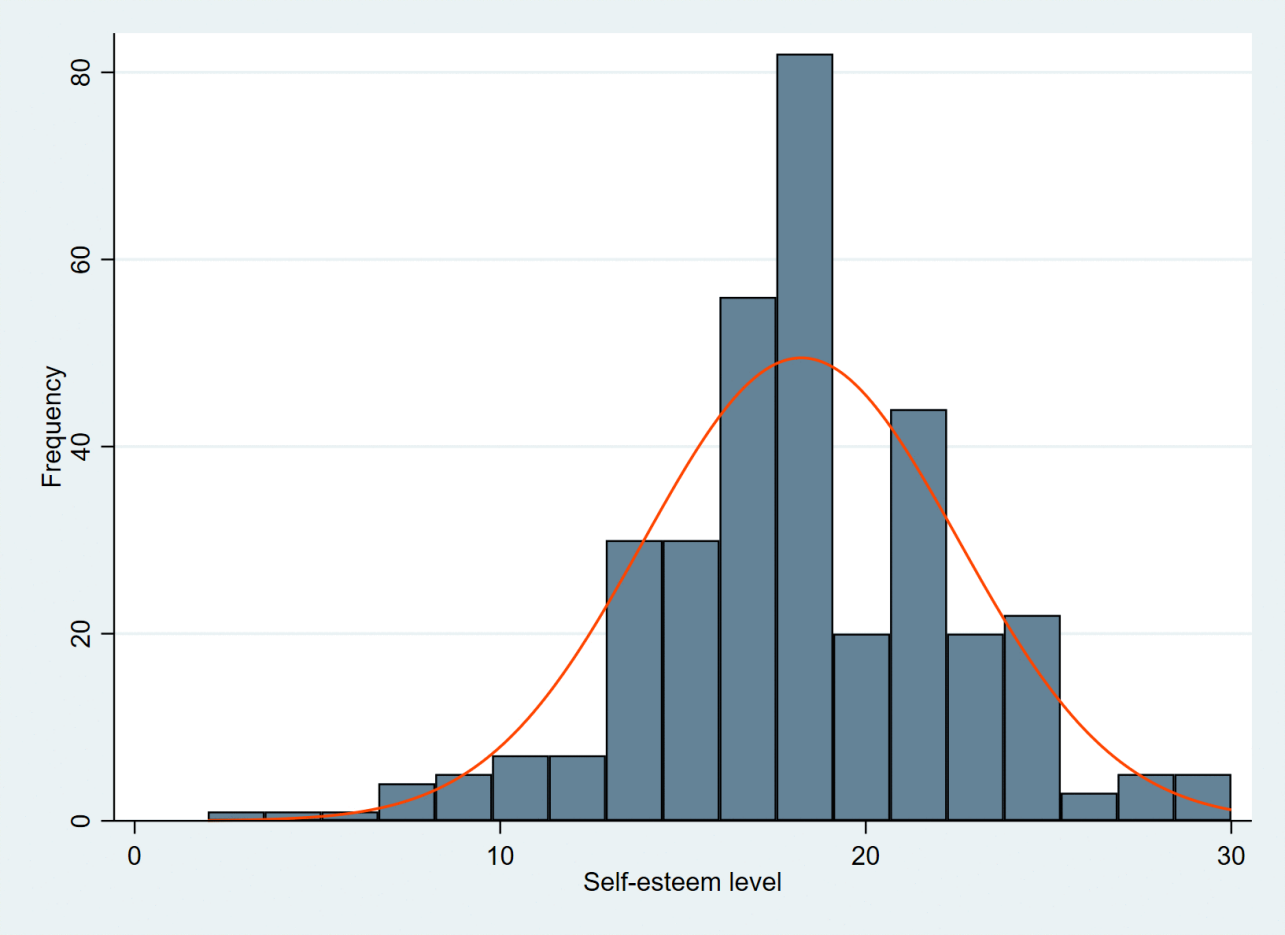
Self-esteem distribution of the study participants.

The histogram depicts the self-esteem level of the study participants. Majority of the participants had moderate-high self-esteem levels with the peak self-esteem score among the participants being around 18. The histogram appears right skewed which indicates that relatively lower proportion of adolescents had a very low level of self-esteem (Fig 1).

Histogram displaying the frequency distribution of self-esteem scores among 343 study participants, measured using the Rosenberg Self-Esteem Scale (RSES).

### Association between self-esteem and perceived parenting styles

The result showed that there was a moderate positive correlation (r = 0.33) between authoritative parenting style and self-esteem. Contrary to this there was a moderate negative correlation (r = −0.32) between authoritarian parenting style and self-esteem, also a moderate negative correlation (r = −0.25) was seen between permissive parenting style and self-esteem (Table 3).

**Table 3:** Association between perceived parenting styles and self-esteem (n = 343).

	Self-esteem	p-value
Authoritative	0.33**	<0.01
Authoritarian	−0.32**	<0.01
Permissive	−0.25**	<0.01

**Correlation is significant at the 0.01 level (2-tailed).

### Factors associated with low self-esteem

Every additional increase in age by one year was associated with 0.7 times lower odds of having low self-esteem (aOR: 0.7; 95% CI: 0.5–0.9). The likelihood of low self-esteem among the adolescents going to private schools was 2.1 times higher than those going to public schools for education (aOR: 2.1;95% CI: 1.1–4.2). Similarly, the likelihood of low self-esteem among the adolescents with father’s education level from illiterate to primary level was 3.8 times higher than those who had secondary level and higher educational level (aOR: 3.8; 95% CI: 1.9–7.4).

A total of 335 observations were included in the multiple logistic regression model. The variables that did not contribute significantly (p>=0.1) were iteratively removed from the analysis based on Wald test. Firstly, the multicollinearity among independent variables included in the model was assessed and there wasn’t any evidence of multicollinearity among the variables (mean VIF = 1.09, all VIFs < 5). The final model had a pseudo-R square value of 8.58%, which indicates that 8.58% variation in the outcome variable, i.e., low self-esteem problem was explained by all the independent variables [age, father’s education level, school type, family type] in the model. According to the likelihood ratio test (LR χ² = 25.66, p < 0.05), all the independent variables in the model were significantly predictive of the low self-esteem, i.e., the proposed model is better than the null model. The Hosmer–Lemeshow goodness-of-fit test produced a p-value of 0.14, suggesting that the model provides a good fit, with observed and predicted values showing close agreement in predicting low self-esteem (Table 4).

**Table 4:** Factors associated with low self-esteem (n = 335).

Independent Variables	Unadjusted Odds ratio (95% CI)	Adjusted Odds ratio (95% CI)	P-value
Age	0.7 (0.5-0.9)	0.7 (0.5-0.9)	0.01
School Type			
Private	1.8 (0.9-3.2)	2.1 (1.1-4.2)	0.03
Public			Ref
Father’s Education Level			
Less than secondary	2.3 (1.3-4.2)	3.8 (1.9-7.4)	0.00
Secondary and above			Ref
Family Type			
Nuclear	1.8 (0.9-3.2)	1.7 (0.9-3.1)	0.10
Joint/ Extended			Ref

^a^Pseudo R^2^= 0.0858, p=0.0000, LR Chi2 (5) =25.66.

## Discussion

Our study revealed that one out of six (16.3%) adolescents had low self-esteem which is consistent with the similar studies in urban areas of Nepal, that reported low self-esteem rates ranging from 21% to 29% [10–13]. The possible explanation behind the high prevalence of low self-esteem among adolescents could be due to major developmental changes in adolescence like puberty, major body and hormonal changes, identity development which are coupled with increased peer pressure, academic stress, social media comparisons and changing family dynamics.

Our study found that self-esteem improves with age, consistent with the findings from a study in Switzerland [29]. The possible explanation behind this could be because younger adolescents undergo rapid and noticeable physical changes during puberty, which can lead to confusion or dissatisfaction with their bodies. According to Harter’s Body Image theory,these changes—such as height, weight, and secondary sexual characteristics—often lower self-esteem. Early adolescents may feel awkward or self-conscious, whereas older adolescents typically adjust to these changes. This transition highlights the dynamic nature of self-evaluation during early adolescence regarding how self-perception evolves over time, particularly through social interactions, self-evaluation, and competence in different domains (academic, social and physical) [30]. On top of this, social changes during early adolescence, such as the shift in social environment that occurs when transitioning to secondary school, can significantly impact self-esteem. Early adolescent period brings changes in academic expectations and behavioral demands, which contributes to the evolving self-evaluation. Additionally, the increasing importance of social comparisons during this stage plays a key role in shaping self-esteem, particularly in areas related to one’s social interactions and context [31]. Although the middle and older adolescence period cannot be completely ruled out with regards to changes in the physical, social and academic areas, the increasing maturity of adolescents and decreasing maturity gap may act as protective factors that limit the fluctuation of self-esteem. Erik Erikson’s theory of psychosocial development suggests that adolescents go through the stage of ‘Identity vs. Role Confusion’. While younger adolescents are still figuring out who they are, leading to uncertainty and lower self-esteem; older adolescents, develop a clearer sense of identity, and are more confident and better equipped to handle challenges, resulting in higher self-esteem compared to their younger counterparts [32].

Another significant association was observed between type of school and self-esteem of the participants in our study. Students attending private schools were found to have lower self-esteem compared to those attending public schools. The reason behind this could be the relatively higher academic stress among private school students [33] and greater academic competition among students to perform better in private schools whereas in public schools such pressure and competition is lesser [34]. A similar finding has been reported in a study from Bangladesh [35]. This similarity could possibly be explained by the reason that public schools in Bangladesh have skilled and professionally sound teachers, better student-teacher ratios, more educational equipment and aids, a substantial financial allocation from the government, better admission procedure etc., causing its effects on private schools, which has recently been the same case in Nepal as better infrastructures, teacher availability and skilled teachers, better programs and interventions targeted mostly to public schools have created an enabling environment for better mental health and self-esteem enhancement of adolescent students in public schools.

In the present study, low self-esteem was significantly associated with father’s education level. Adolescents whose fathers had at least a secondary education level, scored higher in self-esteem than those whose fathers had less than secondary education, consistent with findings from a Turkish study [36]. Nepal’s patriarchal society positions the father as the central family figure, making his influence crucial in shaping his children’s self-perception. Education equips fathers with parenting knowledge, communication skills, higher income, and social prestige. It also enhances their understanding of their children’s psychological needs and effective coping strategies during critical life stages.

Our study concluded that the practice of authoritative parenting showed a significant positive association with self-esteem level of the participants, compared to other two parenting styles. This is because parental involvement in their children’s affairs, the acceptance from parents, their support and having a good inter-relationship with their ward are beneficial for their children’s overall self-esteem. Several other past studies conducted in the United States, Hungary and Spain have also highlighted the benefits of authoritative parenting [19–21]. A meta-analysis conducted in this topic also reported that adolescents who perceived that their parents’ parenting style as authoritative type, had a significantly higher likelihood of possessing a higher self-esteem level [37]. Same finding also applied for the previous studies conducted in urban Nepal settings [10,13]. A recent study from a rural district of Nepal, however, reported findings that contradicted our results by finding a negative association between authoritative parenting and adolescents’ self-esteem [38]. This discrepancy may be explained by contextual differences between the study settings, particularly urban versus rural socio-cultural environments. Our study was conducted in one of the most socioeconomically developed and urbanized regions of Nepal, where parents generally have higher levels of education, greater access to parenting-related information, and more opportunities for parent–school engagement. These factors may enable urban parents to practice authoritative parenting in a way that is more balanced—combining warmth, involvement, and support with appropriate expectations and consistent boundaries. Although another study from Brazil highlighted that permissive parenting contributed more or equal to the self-esteem of adolescents compared with authoritative parenting [22], permissive parenting did not show a beneficial effect on the self-esteem of Nepalese adolescents in our study. The possible explanation behind this could also be the difference in parental understanding of the concepts of different parenting styles. Also, Brazil is often termed as a horizontal collectivist society, where non-hierarchical relationships and egalitarianism are into practice. Therefore, permissive parenting, which is interpreted as low strictness and high acceptance, may be perceived by individuals as supportive of self-growth and autonomy, while additional parental strictness or expectations from may not be beneficial for their self-esteem development. In contrast, Nepal, which is a vertical collectivist country; hierarchy, discipline, obedience, guidance and behavior direction are normally expected between relationships as normative. Within this framework, parental strictness and expectations may be interpreted as care, guidance, and responsible involvement, whilst low-control approaches may be perceived by the offsprings as negligence and lack of affection [22].

The study’s findings flag the necessity to minimize the practice of authoritarian parenting style. In place of relying on negative criticism and punishment, parents must aim to address any misbehavior of their ward through positive communication, mutual understanding, and fostering a supportive, non-threatening environment. Even though, during adolescence years of people’s walk of their lives, they spend much more time with their peers rather than with their parents and may sometimes openly challenge their parents’ actions and beliefs, still they value their relationships with their parents immensely. Adolescents are always the ones who can still be molded into good human beings by their parents. Therefore, parents should be counseled to help them understand the undeniable important fact that they are the best and most important resources for adolescent’s positive future development.

### Limitations

The findings of this study should be interpreted in light of its limitations. In this study, parenting style and self-esteem were measured using perception-based scales, which could differ from actual parental behavior employed by the parents. Consequently, adolescents with lower self-esteem may be more likely to interpret or perceive parental behaviors in a more negative manner. This raises the possibility of bidirectional influence, where perceptions of parenting may partly reflect the adolescent’s psychological state rather than solely objective parental practices Also, there may have been recall biases, albeit sufficient probing was done by data collectors to minimize this bias. This study was conducted in urban high schools; therefore, the findings would not apply to the understanding of adolescents and parents living in rural areas and to adolescents who do not attend formal schools. A self-report measure was used for data collection and self-report measures are subjected to socially desirable responses. This was tried to be reduced by conducting the data collection by providing enough private space for everyone and by informing the participants that their responses were de-identified. Although we adjusted for key socio-demographic variables, there may be potential confounding from other factors such as peer relationships, parental income, or parental mental health. These factors could influence both adolescents’ perception of parenting style and their self-esteem, potentially biasing the observed associations. Future longitudinal studies with more comprehensive measures are needed to better control for these confounders. The participants of this study were nested within schools, which could have potential for intra-cluster correlation. This correlation within cluster was not taken into account by this study. Finally, the cross-sectional nature of this study restricts the ability to infer causal relationships between the identified factors and self-esteem.

## Conclusion

This study revealed that 16.3% of school adolescents in Tokha municipality have a low self-esteem level. Main factors that are significantly associated with low self-esteem include the type of school attended, father’s education level and respondent’s age. Similarly, authoritative style of parenting has positive correlation with self-esteem while other two are negatively correlated. Cost effective programs targeting self-esteem improvement, particularly among younger adolescents and in families with lower paternal education, are recommended. Implementing school-based programs that strengthen parents’ knowledge, perceptions, and skills in authoritative parenting may support healthier adolescent development. Further research is warranted to better understand the factors influencing adolescent self-esteem and to guide effective interventions.

## Supporting information

S1 File STROBE checklist. (PDF)

S2 File Operational Definitions. (PDF)

S3 File Deidentified Dataset. (XLSX)

## References

[1] World Health Organization. Mental health of adolescents. 2025. https://www.who.int/news-room/fact-sheets/detail/adolescent-mental-health

[2] World Health Organization. Global Accelerated Action for the Health of Adolescents (AA-HA!): guidance to support country implementation Geneva: 2017.

[3] Masselink M, Van Roekel E, Oldehinkel AJ. Self-esteem in early adolescence as predictor of depressive symptoms in late adolescence and early adulthood: The mediating role of motivational and social factors. J Youth Adolesc. 2018;47(5):932–46. doi: 10.1007/s10964-017-0727-z 28785953 PMC5878202

[4] Jayanthi P, Rajkumar R. Is low self-esteem a risk factor for depression among adolescents? An analytical study with interventional component. Inte Jour of Medi Res & Health Sci. 2014;3(3):627. doi: 10.5958/2319-5886.2014.00408.1

[5] Handren LM, Donaldson CD, Crano WD. Adolescent alcohol use: Protective and predictive parent, peer, and self-related factors. Prev Sci. 2016;17(7):862–71. doi: 10.1007/s11121-016-0695-7 27562038 PMC5902017

[6] Trzesniewski KH, Donnellan MB, Moffitt TE, Robins RW, Poulton R, Caspi A. Low self-esteem during adolescence predicts poor health, criminal behavior, and limited economic prospects during adulthood. Dev Psychol. 2006;42(2):381–90. doi: 10.1037/0012-1649.42.2.381 16569175

[7] Enejoh V, Pharr J, Mavegam BO, Olutola A, Karick H, Ezeanolue EE. Impact of self esteem on risky sexual behaviors among Nigerian adolescents. AIDS Care. 2016;28(5):672–6. doi: 10.1080/09540121.2015.1120853 26674246 PMC4972583

[8] Banstola RS, Ogino T, Inoue S. Self-esteem, perceived social support, social capital, and risk-behavior among urban high school adolescents in Nepal. SSM Popul Health. 2020;11:100570. doi: 10.1016/j.ssmph.2020.100570 32258358 PMC7115101

[9] World Health Organization. Mental health status of adolescents in South-East Asia: Evidence for action. New Delhi: World Health Organization. 2017.

[10] Banstola RS, Ogino T, Inoue S. Impact of Parents’ knowledge about the development of self-esteem in adolescents and their parenting practice on the self-esteem and suicidal behavior of urban high school students in Nepal. Int J Environ Res Public Health. 2020;17(17):6039. doi: 10.3390/ijerph17176039 32825158 PMC7504235

[11] Paudel S, Adhikari C, Chalise A, Gautam H. Factors associated with self-esteem among undergraduate students of Pokhara metropolitan, Nepal: A cross-sectional study. Europasian Journal of Medical Sciences. 2020;2:98–105. doi: 10.46405/ejms.v2i2.189

[12] Poudel K, Dangol BK, Shrestha R. Mental health problems and self-esteem among the schoolchildren of secondary school in Dharan. J Adv Acad Res. 2019;6(1):65–76. doi: 10.3126/jaar.v6i1.35365

[13] Malla S, Bajracharya S, Shrestha S. Perceived parenting style and self-esteem among adolescents of a secondary school. Journal of Chitwan Medical College. 2024;14(4):58–65. doi: 10.54530/jcmc.1584

[14] Francis A, Pai MS, Badagabettu S. Psychological well-being and perceived parenting style among adolescents. Compr Child Adolesc Nurs. 2021;44(2):134–43. doi: 10.1080/24694193.2020.1743796 32302254

[15] Baumrind D. Current patterns of parental authority. Developmental Psychology. 1971;4(1, Pt.2):1–103. doi: 10.1037/h0030372

[16] Lang D. Parenting Styles. Ames, Iowa: Iowa State University Digital Press. 2020.

[17] Kunwor A, Khadka B, Pariyar M, Ghimire N, Panta P, Baraili S. Exploring parental involvement practices of child rearing in five major cities of Nepal. SXC Journal of Management and Social Sciences. 2021;I(I):33–43.

[18] Bronfenbrenner U, Morris PA. The Bioecological Model of Human Development. Handbook of Child Psychology. Wiley. 2007. doi: 10.1002/9780470147658.chpsy0114

[19] Milevsky A, Schlechter M, Netter S, Keehn D. Maternal and paternal parenting styles in adolescents: Associations with self-esteem, depression and life-satisfaction. J Child Fam Stud. 2006;16(1):39–47. doi: 10.1007/s10826-006-9066-5

[20] Piko BF, Balázs MÁ. Authoritative parenting style and adolescent smoking and drinking. Addict Behav. 2012;37(3):353–6. doi: 10.1016/j.addbeh.2011.11.022 22143001

[21] García F, Gracia E. Is always authoritative the optimum parenting style? Evidence from Spanish families. Adolescence. 2009;44(173):101–31. 19435170

[22] Martínez I, García JF, Yubero S. Parenting styles and adolescents’ self-esteem in Brazil. Psychol Rep. 2007;100(3 Pt 1):731–45. doi: 10.2466/pr0.100.3.731-745 17688087

[23] Gunjan S, Neelam Py. Parenting styles and its effect on self-esteem of adolescents. Int J Indian Psychol. 2015;3(1). doi: 10.25215/0301.114

[24] National Statistics Office. National Population and Housing Census 2021. National Statistics Office, Nepal; 2021. https://censusnepal.cbs.gov.np/results/population?province=3&district=28&municipality=5

[25] Charan J, Biswas T. How to calculate sample size for different study designs in medical research?. Indian J Psychol Med. 2013;35(2):121–6. doi: 10.4103/0253-7176.116232 24049221 PMC3775042

[26] Rosenberg M. Society and the adolescent self-image. Princeton (NJ): Princeton University Press. 1965.

[27] Divya TV, Manikandan K. Perceived Parenting Style Scale. Kerala, India: Department of Psychology, University of Calicut. 2013.

[28] Bennet L, Dahal DR, Govindasamy P. Caste, ethnic, and regional identity in Nepal: further analysis of the 2006 Nepal demographic and health survey. Calverton, Maryland, USA: Macro International. 2008.

[29] Erol RY, Orth U. Self-esteem development from age 14 to 30 years: A longitudinal study. J Pers Soc Psychol. 2011;101(3):607–19. doi: 10.1037/a0024299 21728448

[30] Harter S, Leahy RL. The construction of the self: A developmental perspective. J Cogn Psychother. 2001;15(4):383–4. doi: 10.1891/0889-8391.15.4.383

[31] Wigfield A, Eccles JS, Mac Iver D, Reuman DA, Midgley C. Transitions during early adolescence: Changes in children’s domain-specific self-perceptions and general self-esteem across the transition to junior high school. Developmental Psychology. 1991;27(4):552–65. doi: 10.1037/0012-1649.27.4.552

[32] Palm M. Erikson’s Theory of Psychosocial Development. Baylor University. 2023.

[33] Hussain A, Kumar A, Husain A. Academic stress and adjustment among high school students. Journal of the Indian Academy of Applied Psychology. 2008;34:70–3.

[34] Ghosh SM. Academic stress among government and private high school students. The International Journal of Indian Psychology. 2016;3(2). doi: 10.25215/0302.146

[35] Islam MN. Study habits, self-esteem, and academic achievement among public and private secondary school students in Bangladesh. International Journal of Psychology and Educational Studies. 2022;8(3):39–50. doi: 10.52380/ijpes.2021.8.3.214

[36] Şahin E, Barut Y, Ersanlı E. Parental education level positively affects self-esteem of Turkish adolescents. Journal of Education and Practice. 2013;20:87–97.

[37] Pinquart M, Gerke D-C. Associations of parenting styles with self-esteem in children and adolescents: A meta-analysis. J Child Fam Stud. 2019;28(8):2017–35. doi: 10.1007/s10826-019-01417-5

[38] Khadka R, Bhatt A, Thapa M, Sharma A, Joshi M, Mishra DK. Relationship of parenting styles on depression, anxiety, stress and self-esteem of adolescents. PLoS One. 2025;20(12):e0332854. doi: 10.1371/journal.pone.0332854 41335587 PMC12674523

