# Supplementary material for: Influence of parenting style on the self-esteem of adolescents and the factors associated with low self-esteem: An institutional based cross-sectional study in Tokha municipality, Nepal
Source: PLoS One. 2026 May 13;21(5):e0347664. doi: 10.1371/journal.pone.0347664 (PMC13170850; doi:10.1371/journal.pone.0347664)
Supplement: S1 File — (PDF) [file pone.0347664.s001.pdf]

STROBE Statement—Checklist of items that should be included in reports of ***cross-sectional studies***

	Item No	Recommendation	Page No
**Title and abstract**	1	(*a*) Indicate the study's design with a commonly used term in the title or the abstract<br>*Cross- sectional study has been mentioned in the title.*	1
		(*b*) Provide in the abstract an informative and balanced summary of what was done and what was found<br>*The abstract part summarizes all the methods employed and the main findings.*	1-2
**Introduction**			
Background/rationale	2	Explain the scientific background and rationale for the investigation being reported<br>*All the scientific explanation along with rationale for the study being undertaken has been described under the heading "Introduction".*	2-3
Objectives	3	State specific objectives, including any prespecified hypotheses<br>*The objective of the study has been clearly specified in the "Background" section's last paragraph.*	3
**Methods**			
Study design	4	Present key elements of study design early in the paper<br>*The study design has been clearly stated under the sub-section "Study design" of the "Materials and methods" section.*	3
Setting	5	Describe the setting, locations, and relevant dates, including periods of recruitment, exposure, follow-up, and data collection<br>*All the relevant information such as settings/location, data collection has been clearly mentioned under the sub-section "Setting of the Study".*	3
Participants	6	Give the eligibility criteria, and the sources and methods of selection of participants<br>*All the relevant information pertaining to eligibility criteria, sources and methods of selection of participants has been clearly mentioned under the subsection "Study Participants" and "Sampling Technique and Sample Size".*	3
Variables	7	Clearly define all outcomes, exposures, predictors, potential confounders, and effect modifiers. Give diagnostic criteria, if applicable<br>*A separate file for operational definition has been attached.*	
Data sources/ measurement	8*	For each variable of interest, give sources of data and details of methods of assessment (measurement). Describe comparability of assessment methods if there is more than one group.<br>*All the dependent and independent variables have been mentioned in the sub-section "Measurements", their measurement process and sources are clearly stated under the sub-section "Data collection tools and technique".*	*4*

Bias	9	Describe any efforts to address potential sources of bias<br>*The possibility of recall and social desirability biases was addressed in our study. Social desirability bias was minimized by informing the participants about de-identification process whereas recall bias was addressed through enough probing.*	10
Study size	10	Explain how the study size was arrived at<br>*Sample size determination is clearly stated under the sub-section “Sampling technique and sample size" of method section.*	3
Quantitative variables	11	Explain how quantitative variables were handled in the analyses. If applicable, describe which groupings were chosen and why<br>*All the process employed to analyse the quantitative variable is clearly discussed under the sub-section "Data management and statistical analysis" of materials and method section.*	4-5
Statistical methods	12	Describe all statistical methods, including those used to control for confounding.<br>*Statistical methods are discussed under the sub-section "Data management and statistical analysis".*	4-5
		(*b*) Describe any methods used to examine subgroups and interactions<br>*Described under the sub-section“Data management and statistical analysis". Dichotomization and dividing variables into more than two levels were based on external criteria, extensive literature review and standard operationalization.*	4-5
		(c) Explain how missing data were addressed<br>*Possible checks were employed by extensive monitoring by the investigators during data collection to avoid any missing and errors. Missed data points were collected on the other day by looking into the records of study participants (hard copy). Those participants whose data were ambiguous were excluded from the analysis. Observation with missed values for most of the variables were deleted whereas those with missed variable for only few variables were included in the analysis.*	
		(*d*) If applicable, describe analytical methods taking account of sampling strategy.<br>*All the process for sampling strategy is briefed under the sub-section "Sampling technique and sample size".*	3
		(e) Describe any sensitivity analyses<br>.	

**Results**			
Participants	13*	(a) Report numbers of individuals at each stage of study—eg numbers potentially eligible, examined for eligibility, confirmed eligible, included in the study, completing follow-up, and analysed.<br>*The estimated sample size calculated, and the actual sample collected has been mentioned under the subsection "Sampling technique and sample size".*	3-4
		(b) Give reasons for non-participation at each stage<br>*NA*	
		(c) Consider use of a flow diagram<br>*N/A*	
Descriptive data	14*	(a) Give characteristics of study participants (eg demographic, clinical, social) and information on exposures and potential confounders<br>*Socio-demographic characteristics of the study participants is clearly presented in Table 1.*	6
		(b) Indicate number of participants with missing data for each variable of interest	
Outcome data	15*	Report numbers of outcome events or summary measures<br>*Low self-esteem is the primary outcome variable for the regression analysis and correlation analysis. Both numbers and percentage/proportion are reported throughout the result section.*	5-7
Main results	16	*(a)* Give unadjusted estimates and, if applicable, confounder-adjusted estimates and their precision (eg, 95% confidence interval). Make	

Variables	***Missing***	***Total***	***Percent Missing***
Age	*0*	*343*	*0.00*
Sex	*0*	*343*	*0.00*
Ethnicity	*0*	*343*	*0.00*
Religion	*0*	*343*	*0.00*
Family type	*0*	*343*	*0.00*
Parents' marital status	*0*	*343*	*0.00*
Highest level of father's education	*8*	*343*	*2.33*
Highest level of mother's education	*2*	*343*	*0.58*
Authoritative Parenting style	*0*	*343*	*0.00*
Authoritarian Parenting style	*0*	*343*	*0.00*
Permissive Parenting style	*0*	*343*	*0.00*
Self-esteem level	*0*	*343*	*0.00*
GPA	*8*	*343*	*2.33*

		clear which confounders were adjusted for and why they were included.<br>*The unadjusted odds ratio for the univariate analysis has not been presented in the table. However, the adjusted odds ratio for the multivariable regression analysis has been presented in Table 4.*	
		(b) Report category boundaries when continuous variables were categorized.<br>*Continuous variables like self-esteem (low and medium-high), age, GPA and ethnicity have been categorised based on the external criteria.*	
		(c) If relevant, consider translating estimates of relative risk into absolute risk for a meaningful time period.<br>*Not applicable*	
Other analyses	17	Report other analyses done—eg analyses of subgroups and interactions, and sensitivity analyses<br>*Descriptive/bivariate analysis with appropriate statistical test (Chi-Square test, Fischer's exact test) has been presented in Table 1.Spearman rank correlation has been presented in Table 2. Multiple regression analysis) has also been presented in Table 4..*	
**Discussion**			
Key results	18	Summarise key results with reference to study objectives<br>*Key results with reference to objective are briefly described under the discussion section.*	
Limitations	19	Discuss limitations of the study, taking into account sources of potential bias or imprecision. Discuss both direction and magnitude of any potential bias.<br>*The limitation of the study has been described under the section discussion, in the last paragraph. Potential biases such as recall bias and social-desirability bias may have existed. However, the bias was minimized through probing and using de-identified data and providing ample space to participants for their privacy during data entry. Respectively.*	
Interpretation	20	Give a cautious overall interpretation of results considering objectives, limitations, multiplicity of analyses, results from similar studies, and other relevant evidence.<br>*This has been well described in discussion and conclusion section.*	
Generalisability	21	Discuss the generalisability (external validity) of the study results.<br>*The extent of generalisability (external validity) of the study results is well discussed in the "Limitations" section.*	
**Other information**			
Funding	22	Give the source of funding and the role of the funders for the present study and, if applicable, for the original study on which the present article is based.<br>*This study has not been funded by any organization.*	
