# Supplementary material for: Influence of parenting style on the self-esteem of adolescents and the factors associated with low self-esteem: An institutional based cross-sectional study in Tokha municipality, Nepal
Source: PLoS One. 2026 May 13;21(5):e0347664. doi: 10.1371/journal.pone.0347664 (PMC13170850; doi:10.1371/journal.pone.0347664)
Supplement: S2 File — (PDF) [file pone.0347664.s002.pdf]

Operational Definition

Variables	Description	Categorization/Response Option
**1. Age**	It refers to the completed age of the study participant in years at the time of interview, which was based on self-report. It was measured on a continuous scale.	1. Continuous category
**2. Sex**	Sex was categorized as male and female.	1. Male<br>2. Female
**3. School Type**	It refers to the type of school participants were studying in. It was categorized as public and private.	1. Public<br>2. Private
**4. Parents' Marital Status**	This variable referred to the marital status of respondent's parents at the time of the survey.	1. Married and living together<br>2. Married but not living together<br>3. Divorced<br>4. Separated<br>5. Widow/Widower
**5. Ethnicity**	Based on Health Management Information System (HMIS) classification scheme of Department of Health Services, ethnicity of the study participants was classified as upper caste group (Brahmin and Chhetri), Dalit, Janajati, Madhesi, Muslim and Others.	1. Brahmin and Chhetri<br>2. Janajati<br>3. Dalit<br>4. Madhesi<br>5. Muslim<br>6. Others
**6. Religion**	Religion is this study has been dichotomized, due to low representation of some religion, (such as Muslim, Buddhist, Christian), where Hindu being majority.	1. Hindu<br>2. Non-Hindu
**7. Family Type**	It referred to whether the study participant stayed in a nuclear, joint or extended family.<br><br>Family type was dichotomized due to low percentage of participants belonging to joint and extended families.	1. Nuclear<br>2. Joint<br>3. Extended

**8. Highest level of father's education**	Highest level of father's education was measured using this variable. It refers to the level of completed education achieved by a study participant's father at the time of interview. Illiterate refers to not being able to read and write. Literate refers to being able to read and write (but not formal schooling).	1. Illiterate<br>2. Literate (No formal education)<br>3. Primary level (1-5)<br>4. Secondary level (6-10)<br>5. Higher Secondary level (11-12)<br>6. University level and above (Bachelors and above)
**9. Highest level of mother's education**	Highest level of mother's education was measured using this variable. It refers to the level of completed education achieved by a study participant's mother at the time of interview. Illiterate refers to not being able to read and write. Literate refers to being able to read and write (but not formal schooling).	1. Illiterate<br>2. Literate (No formal education)<br>3. Primary level (1-5)<br>4. Secondary level (6-10)<br>5. Higher Secondary level (11-12)<br>6. University level and above (Bachelors and above)
**7. GPA**	GPA referred to the grade point average scored by the participants in their most recent final exams before the time of the survey.<br><br>In accordance with the National Examination Board (NEB), GPA was categorized dichotomously into "below good" and "good and above" categories.	1. Below good (<2.40)<br>2. Good and above (≥2.40)
**8. Perceived Parenting styles**	This was the parenting style adopted by respondent's parents as perceived by them. There are three parenting styles classified by Baumrind. There were three different Likert scales used for the measurement of these parenting styles separately,<br><br>All three parenting styles were measured as continuous variables without any categorization of which scale the parenting style fell on because of the fact that parenting styles can be combinations of these three styles on some extent rather than explicitly falling on only one parenting style category. The Likert scales of each parenting styles were scored on a scale of 1 to 30 [1].	

**9. Self-esteem**	Self-esteem was defined as the subjective appraisal of the participant's own self-worth by themselves. This referred to how highly or lowly they perceived themselves.<br><br>Self-esteem was measured as a continuous variable using Rosenberg's self-esteem scale which was scored on a level of 1 to 30 [2]. For the analysis, later on the self-esteem was dichotomized into two categories namely "low self-esteem" and "medium-high self-esteem", taking the cut-off value as 15 according to the available literature [3].	1. Low self-esteem (<15)<br>2. Medium-high self-esteem (≥15)

References:

1. Divya, T. V., & Manikandan, K. (2013). Perceived Parenting StyleScale. Department of Psychology, University of Calicut, Kerala, India.
2. Rosenberg, M. (1979). Conceiving the Self. New York: Basic Books.
3. Banstola, R. S., Ogino, T., & Inoue, S. (2020). Impact of Parents' Knowledge about the Development of Self-Esteem in Adolescents and Their Parenting Practice on the Self-Esteem and Suicidal Behavior of Urban High School Students in Nepal. *International Journal of Environmental Research and Public Health*, *17*(17), 6039. https://doi.org/10.3390/ijerph17176039.
